# Shear-Assisted Laser Transfer of Metal Nanoparticle Ink to an Elastomer Substrate

**DOI:** 10.3390/ma11122511

**Published:** 2018-12-11

**Authors:** Wooseop Shin, Jaemook Lim, Younggeun Lee, Sewoong Park, Hyeonseok Kim, Hyunmin Cho, Jaeho Shin, Yeosang Yoon, Habeom Lee, Hyun-Jong Kim, Seungyong Han, Seung Hwan Ko, Sukjoon Hong

**Affiliations:** 1 Optical Nanoprocessing Lab, Department of Mechanical Engineering, Hanyang University, 55 Hanyangdaehak-ro, Sangnok-gu, Ansan, Gyeonggi-do 15588, Korea; caribou11@hanyang.ac.kr (W.S.); limjaemook@hanyang.ac.kr (J.L.); twilit77@hanyang.ac.kr (Y.L.); woong93@hanyang.ac.kr (S.P.); 2 Applied Nano and Thermal Science Lab, Department of Mechanical Engineering, Seoul National University, 1 Gwanak-ro, Gwanak-gu, Seoul 08826, Korea; hs.kim@snu.ac.kr (H.K.); augustinus310@snu.ac.kr (H.C.); jayz.shin84@gmail.com (J.S.); yys1992@snu.ac.kr (Y.Y.); habeom.lee@snu.ac.kr (H.L.); 3 Surface Technology Group, Korea Institute of Industrial Technology, 156 Gaetbeol-ro, Yeonsu-gu, Incheon 21999, Korea; hjkim23@kitech.re.kr; 4 Department of Mechanical Engineering, Ajou University, San 5, Woncheon-Dong, Yeongtong-Gu, Suwon 16499, Korea; sy84han@ajou.ac.kr; 5 Institute of Advanced Machinery and Design (SNU-IAMD), Seoul National University, Gwanak-ro, Gwanak-gu, Seoul 08826, Korea

**Keywords:** laser transfer, metal nanoparticle ink, stretchable electronics

## Abstract

Selective laser sintering of metal nanoparticle ink is an attractive technology for the creation of metal layers at the microscale without any vacuum deposition process, yet its application to elastomer substrates has remained a highly challenging task. To address this issue, we introduced the shear-assisted laser transfer of metal nanoparticle ink by utilizing the difference in thermal expansion coefficients between the elastomer and the target metal electrode. The laser was focused and scanned across the absorbing metal nanoparticle ink layer that was in conformal contact with the elastomer with a high thermal expansion coefficient. The resultant shear stress at the interface assists the selective transfer of the sintered metal nanoparticle layer. We expect that the proposed method can be a competent fabrication route for a transparent conductor on elastomer substrates.

## 1. Introduction

Transparent conducting oxides (TCO) represented by indium tin oxide (ITO) have been the most successful material for transparent conductors, which are becoming increasingly important due to the growing demands in large-area optoelectronics [1,2]. The emergence of flexible and stretchable electronics [3,4], however, has raised a number of issues with these conventional TCOs in terms of their stability towards mechanical disturbances [5] and led to the development of alternative transparent conductors. Among diverse candidates for future transparent conductors [2,3], metal grids with either regular mesh [6,7] or a random percolation network composed of chemically synthesized nanowires [8,9] have been verified to exhibit low sheet resistance and high optical transmittance and to be sufficient for a wide range of optoelectronics, including displays [10,11] and energy devices [12,13]. As a consequence, a number of fabrication techniques, each with their own features, have been developed for the production of various metal grid transparent conductors.

Laser processing is used in micromanufacturing for the fabrication of a wide range of devices, from micromechanical [14] to optical ones [15], and selective laser sintering (SLS) of metal nanoparticle (NP) ink is one of the relevant processes for transparent conductors that enables simultaneous patterning and sintering of metal NP ink for the creation of a metal electrode on either solid or flexible substrates [16]. In a typical procedure, first, metal NP ink is coated on the target substrate, and then the focused laser is scanned over the substrate to increase the local temperature for the selective melting and subsequent coalescence of the NP ink through the photothermal reaction. Being a direct writing method, metal electrodes are created selectively along the scanning path [6,17], and the feature size is mostly determined by the spot diameter of the focused laser, which can be reduced down to even the nanoscale using a tightly focusing lens. Since the fine structures at several micrometers become practically imperceptible to the naked eye, the metal electrodes produced by the SLS of metal NP ink are suitable for transparent conductor applications. At the same time, the minute control of laser parameters permits the application of the SLS process to even the most vulnerable polymer thin films such as polyethylene Terephthalate (PET) [17]. Therefore, various conductive electrodes have been successfully created by the SLS process on flexible substrates with silver (Ag) [6,18], copper (Cu) [19,20] and nickel oxide (NiO) [21] NP ink. On the other hand, the SLS of metal NP ink on elastomer substrates, such as polydimethylsiloxane (PDMS), still remains a challenging task due to the poor wettability of the PDMS substrate together with large discrepancies between the PDMS and the target metals with respect to their mechanical properties [22]. The SLS of metal NP on PDMS has been reported previously by Lee et al. using capillary-assisted laser direct writing (CALDW) [23], but multiple overlapping scanning procedures are required to ensure low sheet resistance, and the resultant metal electrode exhibits relatively large surface roughness, which hinders its application to other optoelectronic devices [24].

In this study, we introduce the shear-assisted laser transfer of metal NP ink to create conductors at the microscale on elastomer substrate by utilizing a large mismatch in thermal expansion coefficients between the elastomer substrate and the metal NP ink layer, which has been considered a problem in other studies [23,25]. The focused laser induces a rapid and confined temperature increase at the focused spot, and the incorporated scanning procedure creates a huge temperature derivative (∂T/∂t) along the scanning path. By conducting the scanning procedure along the metal NP ink sandwiched between the underlying substrate and the target elastomer, shear stress arises at the interface due to the anisotropic thermal expansion and assists the selective transfer of metal NP ink from the donor to the acceptor elastomer substrate. The characteristics of the laser transfer are highly dependent on the laser parameters, including the laser power and the scanning speed, and we confirm that the metal NP ink can be transferred and sintered at the same time in the optimum condition, producing well-defined conductive metal electrodes at microscale on the elastomer substrate with smooth surface morphology.

## 2. Materials and Methods

As a representative metal NP ink, silver (Ag) NP ink was selected and studied throughout the study. Commercial Ag NP ink was purchased (NPS-J, Harima Chemicals, Inc., Tokyo, Japan) and used without any purification. For a typical experiment, Ag NP ink was first deposited on a glass substrate (Microscope slides at 1 mm thickness, Marienfeld, Lauda-Königshofen, Baden-Württemberg, Germany) by a spin-coater (ACE-200, Dong Ah Trade Corp., Seoul, Korea) at 1000 rpm for 400 s. The 532 nm continuous-wave (CW) diode-pumped solid-state (DPSS) laser (Sprout-G-5W, Lighthouse Photonics, San Jose, CA, USA) utilized in this study shows a TEM_00_ beam profile at M^2^ = 1.0–1.1 with a beam diameter of 2.3 mm ± 10%. The PDMS film was separately prepared by initially mixing the resin and agent (Sylgard 184, Dow Corning, Midland, MI, USA) at a 10:1 weight ratio. The PDMS was then coated onto the glass substrate at 100 rpm for 120 s followed by a curing procedure in the oven (OF-12G, JEIO TECH, Daejeon, Korea) at 70 °C for >2 h. The resultant PDMS was carefully attached to the Ag NP ink layer. The laser was focused with a 2× objective lens (M Plan Apo 2×, Mitutoyo, Kawasaki, Japan) on the Ag NP layer, and the sample was scanned using a motorized 2-axis translational stage (ANT130-060-XY-25DU-XY-CMS-MP-PLUS, Aerotech, Pittsburgh, PA, USA) along the programmed scanning path. The laser power and the scanning speed were controlled within the ranges 0.29~1.16 W and 30~250 mm/s, respectively. The transmission and reflection images were taken by an optical microscope (BX53M, Olympus, Tokyo, Japan). Atomic force microscopy (AFM) and scanning electron microscopy (SEM) together with energy dispersive X-ray (EDX) analysis were conducted using NX-10 from Park Systems and JSM-7600f from JEOL (Tokyo, Japan), respectively. 

## 3. Results and Discussion

**Figure 1:**
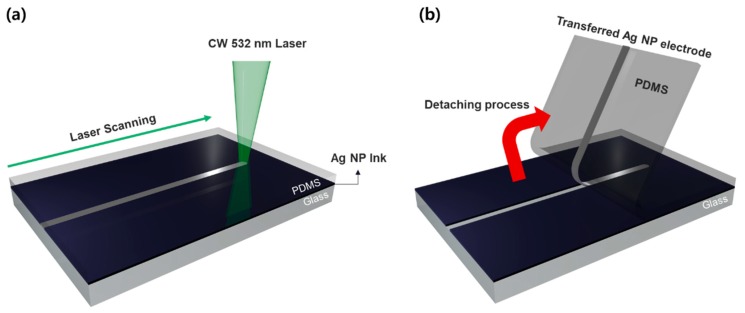
Schematics of the shear-assisted laser transfer of Ag NP ink to the elastomer substrate: (**a**) Laser is focused and scanned on the Ag NP ink, which is sandwiched between the glass substrate and the PDMS film; (**b**) Detaching process after the laser scanning verifies that the Ag NP is selectively sintered and transferred to the PDMS film.

The proposed process is similar to the conventional SLS process for Ag NP ink [6,17]. However, the scanning of the focused laser beam happens at the Ag NP ink layer, which is in conformal contact with the upper PDMS layer as schematically shown in Figure 1a. It is observable that the Ag NP ink is not transferred to the PDMS film spontaneously upon immediate contact, as the Ag NP ink is mostly plasticized after the spin-coating procedure [26]. Since the upper PDMS layer is almost transparent to the CW laser at visible wavelength [27], the photothermal reaction mostly occurs at the Ag NP ink layer, which is sandwiched between the upper PDMS and the underlying glass substrate. The temperature increase at the Ag NP ink layer (∆T) initiates the sintering between the constituent Ag NPs, while the temperature increase in the vicinity leads to the thermal expansion of the resident elements. Due to the large difference in the thermal expansion coefficients, (PDMS: 907 × 10^−6^/K, Ag: 19 × 10^−6^/K, Glass: 4 × 10^−6^/K) [28], we expected that the laser-induced temperature increase as well as its time derivative (∂T/∂t) can create shear stress at the interface, which appears to be critical for the current laser transfer process. In the optimum condition, the sintering of Ag NP and the transfer of the sintered Ag electrode to the PDMS film occurred simultaneously, yet the transfer was not noticeable immediately after the laser scanning procedure. The transferred Ag electrode was verifiable after the detaching the PDMS film (acceptor) from the glass substrate (donor), as shown in Figure 1b. 

**Figure 2:**
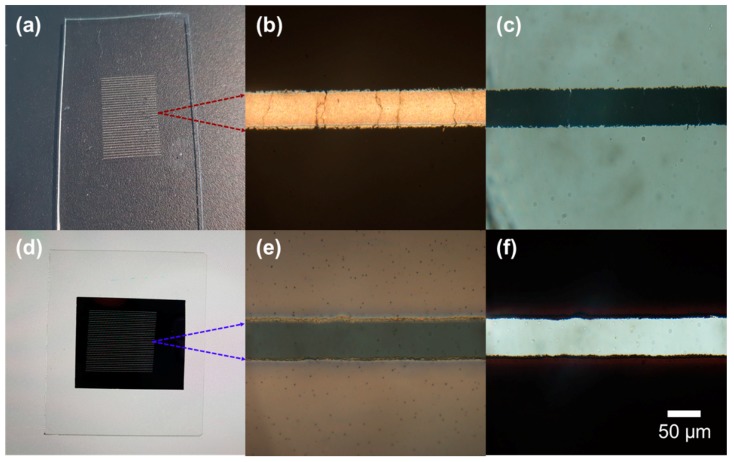
Optical images of (**a**–**c**) PDMS acceptor and (**d**–**f**) Ag NP ink donor on glass substrate; (**a**,**d**) Photographs; (**b**,**e**) Reflection optical microscope images; (**c**,**f**) Transmission optical microscope images.

Upon successful transfer, the effects on the donor and the acceptor should be complimentary: sintered Ag NP ink is selectively created on the acceptor PDMS, while the specific portion of Ag NP ink layer exposed to the scanning laser is removed from the donor glass substrate. The optical images in Figure 2 show the typical transfer results from the macroscopic and microscopic perspectives after conducting the laser scanning along the line patterns at an equal spacing of 250 μm. The optical photographs of the acceptor (Figure 2a) and the donor (Figure 2d) were captured on black and white backgrounds, respectively, in order to display each result more clearly. On the acceptor PDMS, consistent reflective metallic lines are observable both from the optical photograph and the reflection optical microscope image in Figure 2b. The transmission of light, on the other hand, dropped in the corresponding region, as shown in Figure 2c, since the metal layer largely blocked the lights in the visible spectrum owing to its small skin depth. In contrast, white lines were visible on the optical photograph of the donor (Figure 2d) since the light passes through the voids created by the laser transfer. The reflection and transmission microscopic images of the donor in Figure 2e,f can be interpreted likewise. A clear distinction between the laser-scanned and the non-irradiated region ensures the high selectivity of the proposed process. A number of cracks were found on the transferred electrode, which were estimated to have originated during the detaching process, yet the overall electrical conductivity of the sample can be preserved since these nanoscale cracks enable zip-like electrical connections [29,30]. The lateral size of the resultant electrode shown in Figure 2 is at several tens of micrometers. However, the minimum feature size can be reduced simply by using a focusing lens with a higher numerical aperture (NA). For instance, the metal electrode created by a 50× objective lens shown in Supplementary Figure S1 reached the feature size of <5 μm, which is sufficiently small for the application to imperceptible electronics. However, detailed studies were conducted with a low NA objective lens throughout the study, as the higher NA inevitably shrinks the depth-of-focus (DOF) of the focused laser, which in turn narrows the processing window. 

**Figure 3:**
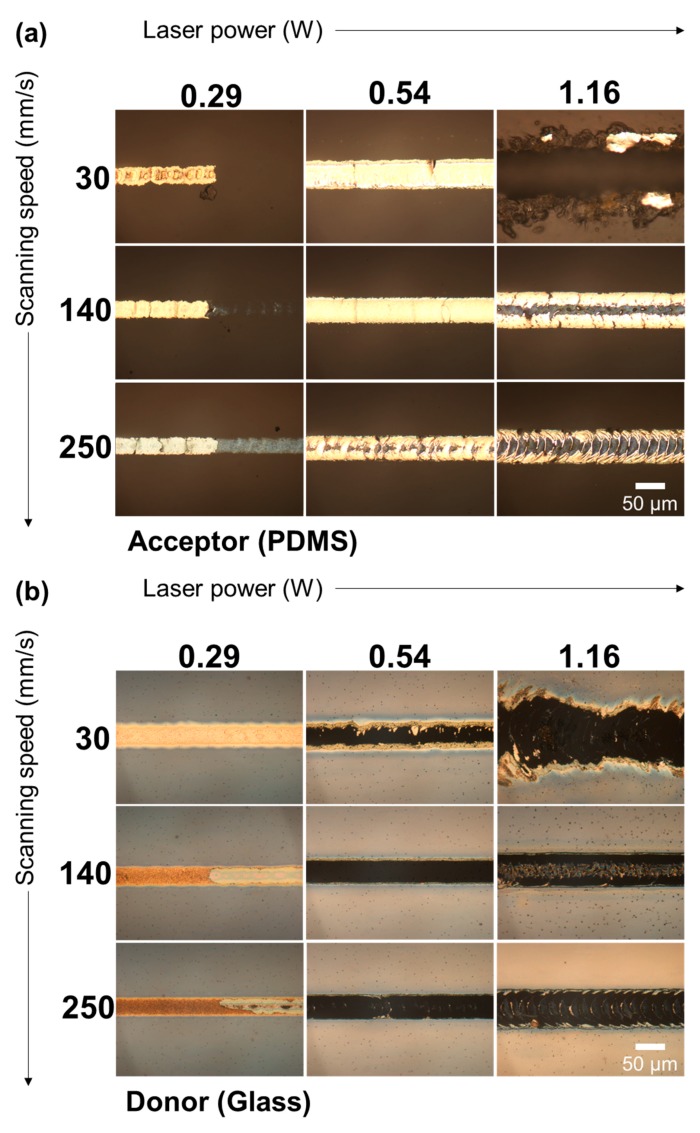
Combinatorial study for optimum laser power vs. laser scanning speed. Reflection optical microscope image of (**a**) the PDMS acceptor and (**b**) the Ag NP layer donor on a glass substrate.

The characteristics of the resultant Ag electrode on the acceptor PDMS, as well as the remaining Ag NP ink on the donor glass substrate, are closely related to the laser power and scanning speed given that the other conditions remain fixed. The reflection microscopic images of the acceptor PDMS and the donor glass substrate from the combinatorial studies on laser power and scanning speed are summarized in Figure 3. Sufficient laser power is assessed to be a prerequisite for effective laser transfer seeing that the transfer rate at 0.29 W power remains to be relatively low at any scanning speed. Nevertheless, the laser power has to be controlled in the moderate range, since the acceptor PDMS can be damaged when the laser fluence exceeds a certain threshold, as representatively shown in the case of 30 mm/s scanning speed with 1.16 W laser power. By increasing the scanning speed, thermal failure at the acceptor PDMS can be prevented even at the same laser power, but the transferred metal electrode suffered from split and detachments (e.g., 1.16 W, 140 mm/s). The transfer probability almost reached unity when the laser was scanned at 140 mm/s with 0.54 W power, but the definition of the transferred Ag electrode became cruder as the scanning speed decreased (e.g., 0.54 W, 30 mm/s). These transfer characteristics are expected to be highly dependent on the time-dependent temperature profile at the Ag NP ink layer and its vicinity, but direct measurement of the temperature distribution was difficult to achieve under the current configuration. Instead, the laser-induced temperature increase, as well as its time derivative, was estimated through theoretical calculation in order to obtain further qualitative insight into the current process. 

**Figure 4:**
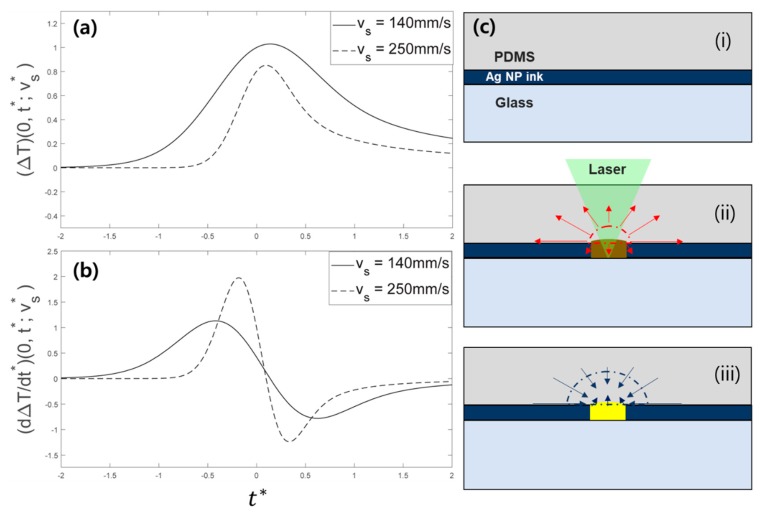
Laser-induced (**a**) temperature increase and (**b**) its gradient observed at a fixed point on the scanning path calculated from the heat equation for two different scanning speeds of 140 mm/s and 250 mm/s; (**c**) Schematics for the laser transfer mechanism: (**i**) Cross-sectional configuration of the system under concern; (**ii**) Laser-induced temperature increase initiates the sintering as well as the expansion of the PDMS layer; (**iii**) Subsequent cooling step causes contraction of the PDMS layer that assists the transfer of the sintered Ag NP.

For the calculation, the heat equation was solved for a semi-infinite substrate with surface absorption under a scanned CW laser beam. The details used for the calculation and the nomenclature can be found in Supplementary Figure S2 [31]. The temperature increase at a fixed point (∆T) and its time derivative (d∆T/dt*) were calculated for two different scanning speeds of 140 mm/s and 250 mm/s for the same laser intensity. Since the absolute value of the temperature increase was not reliable in the current calculation, the temperature evolution was normalized to the maximum temperature increase, which occurred at 140 mm/s, as shown in Figure 4a. It is noticeable that the maximum temperature increase (∆T) became lower at the higher scanning speed, which was easily anticipated since the local heating time reduced at a higher scanning speed. Their time derivatives (d∆T/dt*) shown in Figure 4b, on the other hand, were reversed: a higher scanning speed resulted in a larger maximum temperature derivative. At the same time, each temperature derivative graph shows two peaks in opposite directions, which arose from heating (d∆T/dt* > 0) and cooling (d∆T/dt* < 0), respectively. Seeing that the laser heating and the subsequent cooling induced the expansion and the contraction of the PDMS film in the vicinity, positive and negative d∆T/dt* can be relevant to the shear stress at the Ag NP ink interface in the outward (away from the scanning path) and inward (towards the center of the scanning path) directions.

Based on these features, Figure 4c illustrates the possible mechanism behind the proposed laser transfer. (i) First, the Ag NP ink layer is sandwiched between the underlying glass substrate and the PDMS film. (ii) As the laser passes the point of interest, the laser-induced temperature increase initiates the sintering of the Ag NP ink. At the same time, outward shear stress is applied at the interface together with increasing local pressure due to the thermal expansion of the PDMS film. Our experimental results from Figure 3 suggest that the sintering of Ag NP ink should not be complete at this point, since fully-sintered Ag NP can be split into two by the shear stress, as shown in the microscope image of the 1.16 W, 140 mm/s case. (iii) After the laser-induced temperature increase reaches the maximum, the negative temperature derivative creates inward shear stress. Since the local pressure is decreasing at the same time, the PDMS film under thermal contraction partially enwraps the fully-sintered Ag NP, which is then fastened by the compressive stress. Excessive compressive stress appears to be relaxed by forming a nanoscale wrinkle at the PDMS surface [32], which can often be found in the scanning electron microscope (SEM) images of the PDMS after the laser transfer, as in the attached Supplementary Figure S3. Based on this estimation, the transfer characteristics in Figure 3 can be explained. At 0.29 W laser power and 30 mm/s scanning power, it is observable from the donor side that the Ag NP ink is almost completely sintered (i.e., temperature increase is sufficiently high), but the transfer rate is poor, demonstrating that high ∆T is not a sufficient condition for efficient transfer. By increasing the scanning speed (∆T↓, d∆T/dt*↑), the transfer probability becomes higher, as shown in the corresponding images (e.g., acceptor, 0.29 W, 250 mm/s). From these results, we concluded that the shear force from the rapid temperature increase promoted the transfer of Ag NPs. However, close examination of the donor and the acceptor revealed that the transfer of the Ag NP layer was not complete in this condition: only the upper portion of the Ag NP layer was transferred to the acceptor side. We therefore concluded that sufficient ∆T has to be accompanied with high d∆T/dt* so that the sintering between Ag NPs happens at the same time. The transfer of the Ag NP layer became more complete after a concurrent sintering process since it promoted the adhesion between distinct Ag NPs. An increase in laser power raised the ∆T and d∆T/dt* together. As a result, the transfer rate was able to be enhanced even at a fixed scanning speed (30 mm/s) by increasing the laser power (From 0.29 W to 0.54 W). In the corresponding condition, both ∆T and d∆T/dt* exceeded their respective threshold values and the transfer rate became satisfactory despite the transfer morphology not being optimized, as confirmed from the crude transfer feature. By increasing the scanning speed to 140 mm/s, more impulsive shear force was applied at the Ag NP layer to improve the transfer morphology.

**Figure 5:**
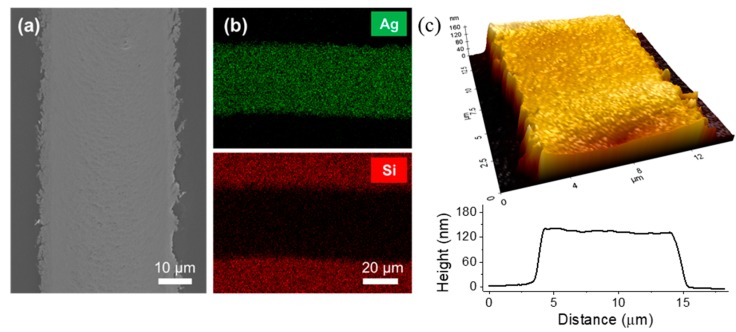
(**a**) SEM, (**b**) EDX and (**c**) AFM measurement of the laser-transferred Ag NP layer on PDMS film.

After the successful laser transfer, the Ag electrode on the PDMS film showed a continuous and smooth morphology, apart from a number of nanoscale zip-like cracks, as shown in the SEM image in Figure 5a. The associated energy dispersive spectrometer (EDS) measurements in Figure 5b further validate that only an insignificant amount of Ag NP ink was transferred from the contact without the laser scanning (refer to Supplementary Figure S4 for pointwise EDS measurements). For the atomic force microscope (AFM) measurement, the laser transfer was conducted with a 5× objective lens instead of a 2× objective lens in order to fit the final Ag electrode line into the scanning range of the AFM. From the AFM profile in Figure 5c, the height was measured to be ~150 nm, which was able to be further controlled by changing the spin-coating conditions. The resultant PDMS film with laser-transferred Ag electrodes may function as a simple form of a detachable electrode. To illustrate this concept, the resultant PDMS was attached to the glass substrate that was partially coated with platinum (Pt), as shown in Supplementary Figure S5. The as-deposited Ag NP ink layer shows negligible electrical conductivity, as shown in Supplementary Figure S5a, since Ag NPs exist as distinct particles. Upon laser transfer, which incorporates the sintering process, the resultant Ag NP exhibited electrical conductance after the laser treatment (Supplementary Figure S5b). Moreover, the PDMS film attached to the glass substrate without any adhesives due to its low mechanical modulus.

## 4. Conclusions

The application of selective laser sintering of metal NP ink on an elastomer substrate has been regarded as a challenging task to date due to the unique mechanical properties of the elastomer substrate. In this study, by having the elastomer substrate in conformal contact with the target metal NP ink layer, we validated that the sintering and the transfer of the metal electrode can happen simultaneously. Our observations suggest that the laser transfer stems from the shear stress exerted by the elastomer, which was determined by the time-dependent temperature profile induced by the scanning laser. The current study concentrated on Ag NP ink and PDMS film as the donor and the acceptor, respectively, yet we anticipate that the proposed laser transfer technique may find use in a wide range of material combinations. Together with the potential of the transferred Ag electrode as a transparent conductor, we expect that the resultant PDMS film will also be an excellent candidate for crack-based sensors, as verifiable from the preliminary studies included in Supplementary Figure S6.

## Supplementary Materials

The following are available online at http://www.mdpi.com/1996-1944/11/12/2511/s1, Figure S1: Reflection optical microscope image of the laser transferred Ag electrode on the PDMS film by 50× objective lens, Figure S2: Schematics for the calculation of temperature rise and its time derivative induced by CW focused laser beam under scanning, Figure S3: SEM image of the wrinkles found in the vicinity of the laser-transferred Ag electrodes, Figure S4: Pointwise EDS measurement on the laser-transferred Ag electrode and the non-irradiated PDMS film, Figure S5: Resistance measurement of Ag NP layer before and after the laser transfer, Figure S6: Experimental setup for the resistance measurement and time-dependent resistance of the transferred Ag microline without mechanical stimuli and under repeated tensile strain at 0.3%.

## References

[1] Gordon R.G. (2000). Criteria for choosing transparent conductors. MRS Bull..

[2] Kwon J., Suh Y.D., Lee J., Lee P., Han S., Hong S., Yeo J., Lee H., Ko S.H. (2018). Recent progress in silver nanowire based flexible/wearable optoelectronics. J. Mater. Chem. C.

[3] McCoul D., Hu W., Gao M., Mehta V., Pei Q. (2016). Recent Advances in Stretchable and Transparent Electronic Materials. Adv. Electron. Mater..

[4] Liu Y., Pharr M., Salvatore G.A. (2017). Lab-on-Skin: A Review of Flexible and Stretchable Electronics for Wearable Health Monitoring. ACS Nano.

[5] Leterrier Y., Médico L., Demarco F., Månson J.A.E., Betz U., Escolà M.F., Kharrazi Olsson M., Atamny F. (2004). Mechanical integrity of transparent conductive oxide films for flexible polymer-based displays. Thin Solid Films.

[6] Hong S., Yeo J., Kim G., Kim D., Lee H., Kwon J., Lee H., Lee P., Ko S.H. (2013). Nonvacuum, Maskless Fabrication of a Flexible Metal Grid Transparent Conductor by Low-Temperature Selective Laser Sintering of Nanoparticle Ink. ACS Nano.

[7] Van de Groep J., Spinelli P., Polman A. (2012). Transparent Conducting Silver Nanowire Networks. Nano Lett..

[8] Lee J.-Y., Connor S.T., Cui Y., Peumans P. (2008). Solution-Processed Metal Nanowire Mesh Transparent Electrodes. Nano Lett..

[9] Lee J., Lee P., Lee H., Lee D., Lee S.S., Ko S.H. (2012). Very long Ag nanowire synthesis and its application in a highly transparent, conductive and flexible metal electrode touch panel. Nanoscale.

[10] Liang J., Li L., Niu X., Yu Z., Pei Q. (2013). Elastomeric polymer light-emitting devices and displays. Nat. Photonics.

[11] Lee J., An K., Won P., Ka Y., Hwang H., Moon H., Kwon Y., Hong S., Kim C., Lee C. (2017). A dual-scale metal nanowire network transparent conductor for highly efficient and flexible organic light emitting diodes. Nanoscale.

[12] Moon H., Lee H., Kwon J., Suh Y.D., Kim D.K., Ha I., Yeo J., Hong S., Ko S.H. (2017). Ag/Au/Polypyrrole Core-shell Nanowire Network for Transparent, Stretchable and Flexible Supercapacitor in Wearable Energy Devices. Sci. Rep..

[13] Lee H., Hong S., Lee J., Suh Y.D., Kwon J., Moon H., Kim H., Yeo J., Ko S.H. (2016). Highly stretchable and transparent supercapacitor by Ag–Au core–shell nanowire network with high electrochemical stability. ACS Appl. Mater. Interfaces.

[14] Athanasiou C.-E., Bellouard Y. (2015). A Monolithic Micro-Tensile Tester for Investigating Silicon Dioxide Polymorph Micromechanics, Fabricated and Operated Using a Femtosecond Laser. Micromachines.

[15] Liu Z., Liao Y., Wang Z., Zhang Z., Liu Z., Qiao L., Cheng Y. (2018). Fabrication of an Optical Waveguide-Mode-Field Compressor in Glass Using a Femtosecond Laser. Materials.

[16] Seung H.K., Heng P., Costas P.G., Christine K.L., Jean M.J.F., Dimos P. (2007). All-inkjet-printed flexible electronics fabrication on a polymer substrate by low-temperature high-resolution selective laser sintering of metal nanoparticles. Nanotechnology.

[17] Yeo J., Hong S., Lee D., Hotz N., Lee M.-T., Grigoropoulos C.P., Ko S.H. (2012). Next Generation Non-Vacuum, Maskless, Low Temperature Nanoparticle Ink Laser Digital Direct Metal Patterning for a Large Area Flexible Electronics. PLoS ONE.

[18] Lee H., Hong S., Kwon J., Suh Y.D., Lee J., Moon H., Yeo J., Ko S.H. (2015). All-solid-state flexible supercapacitors by fast laser annealing of printed metal nanoparticle layers. J. Mater. Chem. A.

[19] Kwon J., Cho H., Eom H., Lee H., Suh Y.D., Moon H., Shin J., Hong S., Ko S.H. (2016). Low-Temperature Oxidation-Free Selective Laser Sintering of Cu Nanoparticle Paste on a Polymer Substrate for the Flexible Touch Panel Applications. ACS Appl. Mater. Interfaces.

[20] Suh Y.D., Kwon J., Lee J., Lee H., Jeong S., Kim D., Cho H., Yeo J., Ko S.H. (2016). Maskless Fabrication of Highly Robust, Flexible Transparent Cu Conductor by Random Crack Network Assisted Cu Nanoparticle Patterning and Laser Sintering. Adv. Electron. Mater..

[21] Lee D., Paeng D., Park H.K., Grigoropoulos C.P. (2014). Vacuum-Free, Maskless Patterning of Ni Electrodes by Laser Reductive Sintering of NiO Nanoparticle Ink and Its Application to Transparent Conductors. ACS Nano.

[22] Trantidou T., Elani Y., Parsons E., Ces O. (2017). Hydrophilic surface modification of PDMS for droplet microfluidics using a simple, quick, and robust method via PVA deposition. Microsyst. Nanoeng..

[23] Ming-Tsang L., Daeho L., Alexander S., Costas P.G. (2011). Rapid selective metal patterning on polydimethylsiloxane (PDMS) fabricated by capillarity-assisted laser direct write. J. Micromech. Microeng..

[24] Yu Z., Zhang Q., Li L., Chen Q., Niu X., Liu J., Pei Q. (2011). Highly Flexible Silver Nanowire Electrodes for Shape-Memory Polymer Light-Emitting Diodes. Adv. Mater..

[25] Xia Y., Whitesides G.M. (1998). Soft Lithography. Angew. Chem. Int. Ed..

[26] Park S., Kwon J., Lim J., Shin W., Lee Y., Lee H., Kim H.-J., Han S., Yeo J., Ko S. (2018). Micropatterning of Metal Nanoparticle Ink by Laser-Induced Thermocapillary Flow. Nanomaterials.

[27] Zahid A., Dai B., Hong R., Zhang D. (2017). Optical properties study of silicone polymer PDMS substrate surfaces modified by plasma treatment. Mater. Res. Express.

[28] Mark J.E. (1999). Polymer Data Handbook.

[29] Kang D., Pikhitsa P.V., Choi Y.W., Lee C., Shin S.S., Piao L., Park B., Suh K.-Y., Kim T.-I., Choi M. (2014). Ultrasensitive mechanical crack-based sensor inspired by the spider sensory system. Nature.

[30] Park B., Lee S., Choi H., Kim J.U., Hong H., Jeong C., Kang D., Kim T.-I. (2018). A semi-permanent and durable nanoscale-crack-based sensor by on-demand healing. Nanoscale.

[31] Bäuerle D. (2011). Laser Processing and Chemistry.

[32] Yang S., Khare K., Lin P.-C. (2010). Harnessing Surface Wrinkle Patterns in Soft Matter. Adv. Funct. Mater..

