# Supplementary material for: Shear-Assisted Laser Transfer of Metal Nanoparticle Ink to an Elastomer Substrate
Source: Materials (Basel). 2018 Dec 11;11(12):2511. doi: 10.3390/ma11122511 (PMC6317006; doi:10.3390/ma11122511)
Supplement: Supplementary file 1 [file materials-11-02511-s001.pdf]

Supporting Information

Shear-Assisted Laser Transfer of Metal Nanoparticle Ink to an Elastomer Substrate

Wooseop Shin [1], Jaemook Lim [1], Younggeun Lee [1], Sewoong Park [1], Hyeonseok Kim [2], Hyunmin Cho [2], Jaeho Shin [2], Yeosang Yoon [2], Habeom Lee [2], Hyun-Jong Kim [3], Seungyong Han [4], Seung Hwan Ko [2,5,*] and Sukjoon Hong [1,*]

[1] Optical Nanoprocessing Lab, Department of Mechanical Engineering, Hanyang University, 55 Hanyangdaehak-ro, Sangnok-gu, Ansan, Gyeonggi-do, 15588, Korea

[2] Applied Nano and Thermal Science Lab, Department of Mechanical Engineering, Seoul National University, 1 Gwanak-ro, Gwanak-gu, Seoul 08826, Korea

[3] Surface Technology Group, Korea Institute of Industrial Technology, 156 Gaetbeol-ro, Yeonsu-gu, Incheon, 21999, Korea

[4] Department of Mechanical Engineering, Ajou University, San 5, Woncheon-Dong, Yeongtong-Gu, Suwon 16499, Korea

[5] Institute of Advanced Machinery and Design (SNU-IAMD), Seoul National University, Gwanak-ro, Gwanak-gu, Seoul 08826, Korea

* Correspondence: maxko@snu.ac.kr (S.H.K.); Tel.: +82-2-880-1681, sukjoonhong@hanyang.ac.kr (S.H.); Tel.: +82-31-400-5249

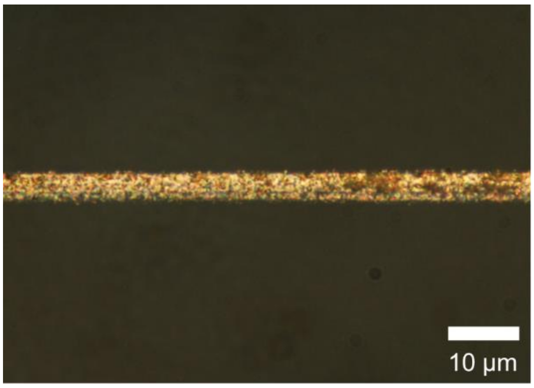

Supporting Figure S1. Reflection optical microscope image of the laser-transferred Ag electrode on the PDMS film by 50 × objective lens, which reaches the feature size of <5 μm.

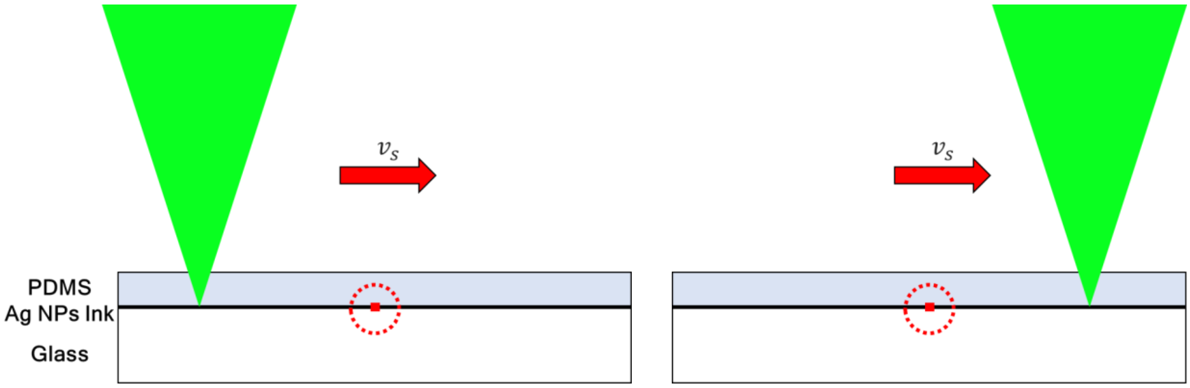

Supporting Figure S2. Schematics for the calculation of the temperature rise and its time derivative induced by CW-focused laser beam under scanning. The following equation has been adopted for the calculation.

$$\Delta T(0, t^*; v_s^*) = \frac{2}{\pi}\theta_c \int_0^\infty \frac{dt_1^*}{t_1^{*\frac{1}{2}}(1+4t_1^*)} \exp(-\frac{v_s^{*2}(t^*-t_1^*)^2}{1+4t_1^*})$$

The equation above corresponds to the following conditions: zero surface conductance (η=0), infinite surface absorption ($\alpha^* = \infty$), characteristic length $l \equiv \omega_0 = 5\ \mu m$. The thermal effect from the upper PDMS film is also excluded. Linearized temperature θ_c is defined as $\theta_c \equiv \theta_c^G \approx 0.89 I_a \omega/\kappa$. The scanning procedure starts at t = $-\infty$ and passes the position x=0 at t=0. The dimensionless velocity is determined from $v_s^* \equiv v_s \omega_0 / D$.

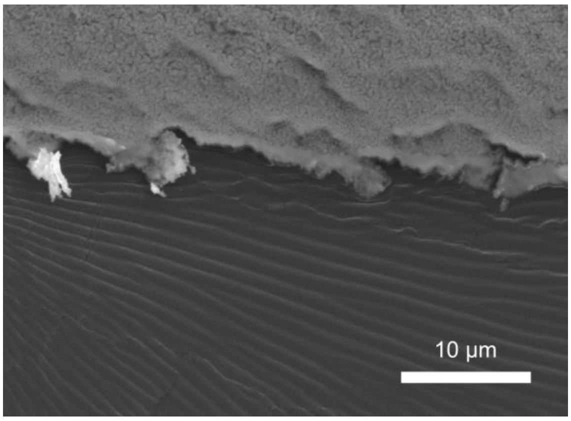

Supporting Figure S3. SEM image of the wrinkles found in the vicinity of the laser transferred Ag electrodes.

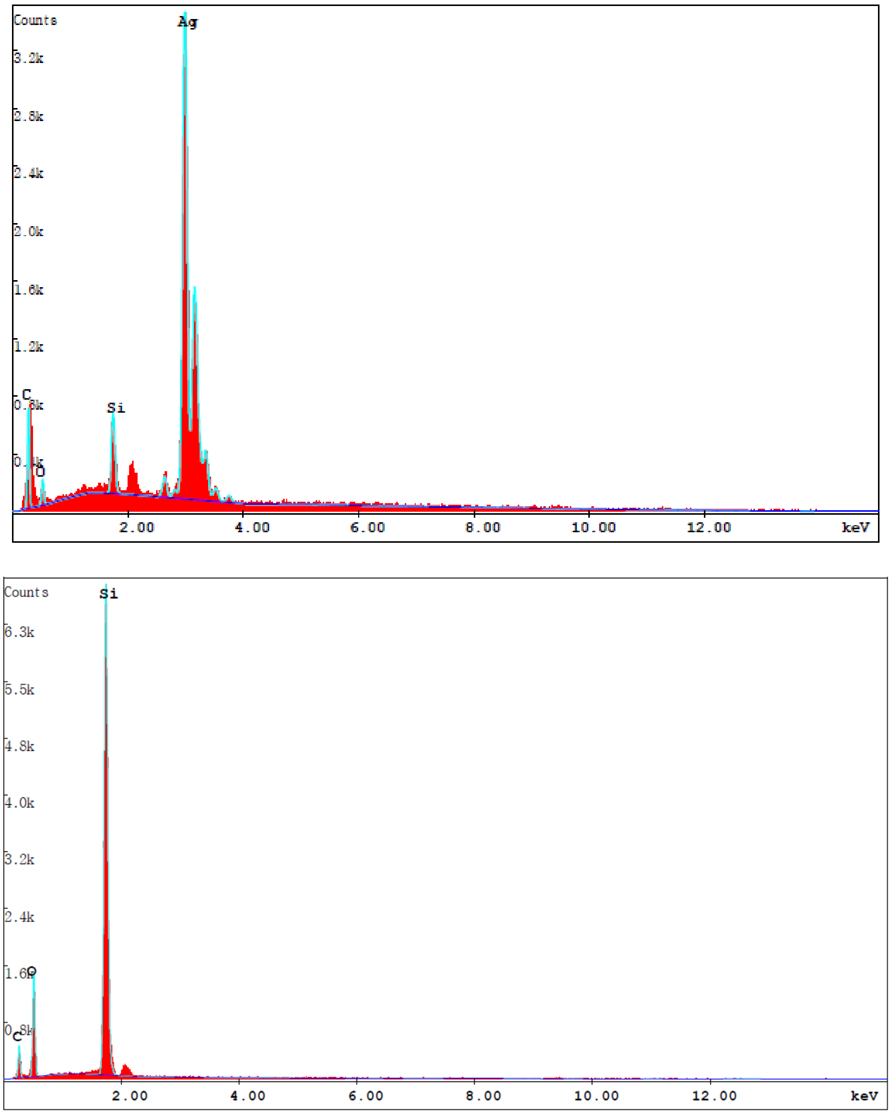

Supporting Figure S4. Pointwise EDS measurement on the laser-transferred Ag electrode and the non-irradiated PDMS film.

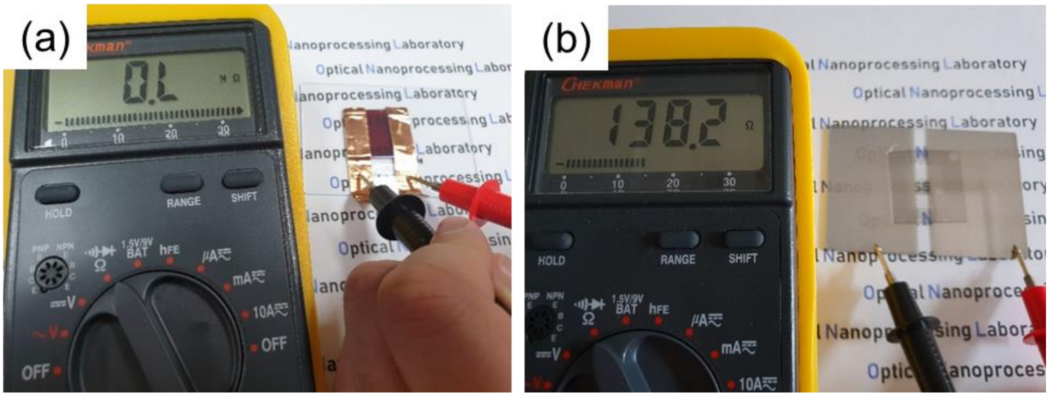

Supporting Figure S5. Resistance measurement of Ag NP layer (a) before and (b) after the laser transfer.

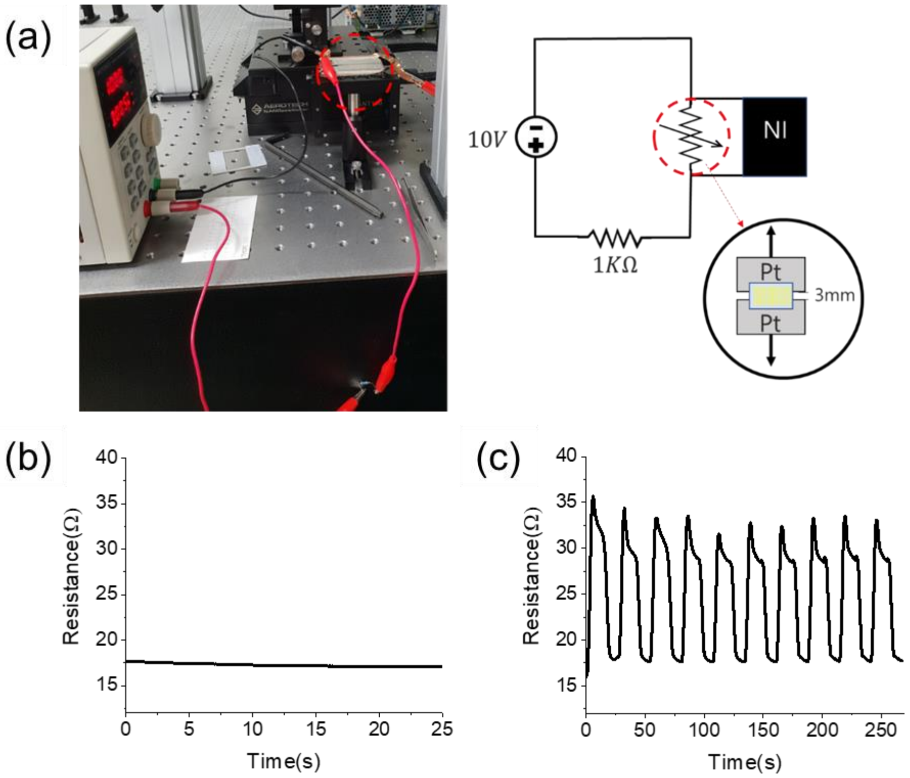

Supporting Figure S6. (a) Experimental setup for the resistance measurement; (b, c) Time-dependent resistance of the transferred Ag microline: (b) without mechanical stimuli; (c) under repeated tensile strain at 0.3 %.
